# Is this an alternative? Time to understand patients’ choices

**DOI:** 10.1016/j.eclinm.2019.09.006

**Published:** 2019-09-27

In the digital era, social media platforms are, for many people, the principal source of information. Although misinformation can spread rapidly and dangerously via these platforms, they are also a powerful source to reach the global population. The recent announcement that Facebook, Instagram, and Pinterest will work with WHO to ensure people can access authoritative information on vaccines, and to reduce the spread of inaccuracies on the topic is therefore good news. This collaboration could help to make life-saving health information more readily available to a much wider population.

Celebrities and other social media influencers can also affect how people perceive information on health. These influencers share many personal aspects of their lives, giving advice and generating trends among their followers. Several complementary and alternative medicine (CAM) practices have gained visibility because famous people endorse them. One example is acupuncture, promoted by movie stars such as Jim Carrey, Sandra Bullock, and Robert Downey Jr. Although the Cochrane Collaboration has concluded that acupuncture might be effective for the treatment of some forms of pain, it highlights that there is no evidence for a benefit in other conditions (such as irritable bowel syndrome, smoking or rheumatoid arthritis) where it is currently used. Among athletes, cupping, whereby rounded inverted cups are briefly attached to parts of the body by means of a vacuum, has gained popularity in the last few years, with swimmer Michael Phelps showing the typical cupping marks during the 2016 Summer Olympics. The procedure of vaginal steaming (v-steam), which involves exposing the vulva to herb-infused steam with the supposed aim of tightening the vagina and freshening it, is another example of a CAM practice. V-steam has been traditionally used in some Asian and African cultures, and in the past few years has become increasingly common in Western and Eastern societies, gaining popularity after endorsement by Gwyneth Paltrow on her popular lifestyle website.

This social media influence is an example of a wider phenomenon: that despite access to evidence-based health care and medication, people sometimes decide, possibly on the basis of inappropriate sources of information, to opt for alternative practices with a poor evidence base, even when their condition is not a one that could benefit from such approaches. These practices, especially if unrelated to the patient's particular medical condition, are not only without beneficial effects, but can also cause harm and adverse reactions. A report by Robert Magali from the University of Calgary (Canada), in the *Journal of Obstetrics and Gynaecology Canada*, draws attention to the harm that such procedures can cause. He describes the case of a woman aged 62 years with a stage IV vaginal prolapse who, advised by a traditional healer, opted for a v-steam instead of surgery. This treatment caused her to develop second-degree burns on her cervix and vagina, which required antibiotic treatment and delayed the necessary surgical interventions.

A systematic review in *Complementary Therapies in Clinical Practice* by Martin R Keene from James Cook University (QLD, Australia) estimated that approximately half of all patients with cancer use CAM remedies alongside conventional cancer treatments. The reasons for doing so are diverse, and range from feeling more in control, to stress relief, to coping with the side-effects of chemotherapy. However, some herbal products might cause adverse reactions, or interact with prescribed drugs, altering their absorption and metabolism, which can, in turn, increase or decrease the effectiveness and toxicity of various drugs. In addition to this, and more concerningly, some people can be convinced that an alternative therapy will cure them when it cannot, and this could lead them to give up their conventional cancer treatment, with very harmful consequences.

The field of CAM is vast and complex, and WHO's 2018 decision to include, for the first time, traditional Chinese medicine (TCM) in its global medical compendium generated a lot of debate amongst physicians and researchers. TCM lays its foundation on the assumption that the body's vital energy (ch'i or qi) circulates through channels called meridians and that, to heal the body, a holistic approach is needed. However, scientific research does not recognise concepts such as qi and meridians. TCM includes several approaches—including acupuncture, cupping therapy, massage, exercise, dietary and herbal therapy—and holds a large share (40 · 3% in 2017) of the Chinese drug market, constituting a strong economic force. Practices such as aromatherapy, herbal infusions, massage, or mindfulness can be useful to relieve stress, and might be beneficial in this regard; however, the requirement of different treatments for different conditions should not be underestimated, and the role of placebo should be taken into account when assessing the efficacy of a given therapy. Moreover, the use of potentially toxic plants or animal parts from endangered species is concerning.

The use of plant-based compounds has ancient roots, and has paved the way for modern pharmacology. Fundamental drugs, such as vinca alkaloids for cancer therapy or artemisinin for malaria treatment, are plant-derived compounds, and TCM holds great potential for the discovery of promising molecules. Additionally, some CAM products probably include naturally active compounds that have yet to be discovered via medicinal chemistry—their effect could just be masked or negated by all the other components.

An interesting perspective by Jeremy Snyder and Timothy Caulfield published this January by *The Lancet Oncology* highlighted another thought-provoking phenomenon: the increase in patients’ crowdfunding campaigns for alternative cancer treatments. Snyder and Caulfield found that patients have three main reasons for seeking CAM treatments: the desire to try every available treatment, fear or scepticism of traditional treatment, and an inability to pursue traditional treatment for financial or medical reasons.

These findings call attention to patients’ needs, and highlight the importance of doctors, researchers, and carers listening and understanding their patients, because their needs probably go beyond treatment and updates on their health. One of the reasons that drives patients to consult traditional practitioners relies on the benefits of touch, talking, and time that a CAM therapist usually offers. Feeling cared for is important in terms of the individual's quality of life, and generates trust and positivity, which can help patients to better cope with their illnesses.

The increasing interest in CAM, often on the basis of inappropriate claims on social media, places a greater imperative on clinical researchers to undertake more thorough investigations into patients’ needs and motivations, and thus gain better understanding of the driving forces guiding their health-related decisions. By ensuring that conventional treatments are reinforced by thoughtful listening to patients’ voices and concerns, we can turn the tide on patients rejecting evidence-based medicine.

*EClinicalMedicine*

